# Preparation and Characteristics of Biocomposites Based on Steam Exploded Sisal Fiber Modified with Amphipathic Epoxidized Soybean Oil Resin

**DOI:** 10.3390/ma11091731

**Published:** 2018-09-14

**Authors:** Bo Lei, Yong Liang, Yanhong Feng, Hezhi He, Zhitao Yang

**Affiliations:** 1 State Key Laboratory of Pulp and Paper Engineering, South China University of Technology, Guangzhou 510640, China; meleibo1991@mail.scut.edu.cn; 2 National Engineering Research Center of Novel Equipment for Polymer Processing, South China University of Technology, Guangzhou 510640, China; pmhzhe@scut.edu.cn; 3 Key Laboratory of Polymer Processing Engineering, Ministry of Education, South China University of Technology, Guangzhou 510640, China; 4 School of Mechanical and Vehicle Engineering, Changzhou Institute of Technology, Changzhou 213032, China; meyongliang@foxmail.com

**Keywords:** screw extrusion steam explosion, epoxidized soybean oil, biocomposites, hydrophobicity, interface bonding

## Abstract

Sisal fiber was pretreated by continuous screw extrusion steam explosion to prepare steam exploded sisal fiber (SESF) preforms. An amphipathic bio-based thermosetting resin with poor mechanical properties was cured by epoxidized soybean oil (ESO) and citric acid (CA). The obtained resin was used to modify SESF preforms and prepare eco-friendly biocomposites. The molar ratios (R) of carboxylic groups to epoxy groups and resin contents in biocomposites were adjusted. The biocomposites were characterized by thermogravimetric analysis (TGA), differential scanning calorimetry (DSC), Fourier-transfer infrared spectroscopy (FT-IR), tensile testing, scanning electron microscopy (SEM), water absorption and water contact angle measurements. The maximum thermal decomposition temperature of the biocomposites was 373.1 °C. The curing efficiency of the resin in the biocomposites improved with the increase of resin content, and reached a maximum at R = 1.2. The tensile strength of the biocomposites reached a maximum of 30.4 MPa at R = 1.2 and 40% resin content. SEM images showed excellent interfacial bonding and fracture mechanisms within the biocomposites. The biocomposites exhibited satisfactory water resistance. ESO resin cured with polybasic carboxylic acid is therefore a good bio-based modifier for lignocellulose, that prepare biocomposites with good mechanical properties, hydrophobicity, and thermostability, and which has a potential application in packaging.

## 1. Introduction

The widespread use of petroleum-based materials and products is leading to depleted nonrenewable resources and increased environmental pollution. This has prompted research on environmentally friendly substitutes for these materials. Lignocellulose is one of the most abundant and renewable natural resources, and can be obtained from wood, herbage, crop straw, hemp, and bagasse, etc. Lignocellulose is frequently applied in composites as a reinforcing element [1], or is converted into biomass, such as fuels and chemical products [2]. However, the recalcitrant structure of lignocellulose requires cumbersome pretreatment processes, which result in much of the lignocellulose not being fully utilized. Most lignocellulose is used to produce low value packaging materials, such as lignocellulose board [3], molded pulp [4], and wood plastic composites (WPCs) [5]. Most of these materials have minimal environmental impact, as they are biodegradable, but their poor water resistance and mechanical properties limit their application lifespan.

Much recent attention has focused on developing biocomposites composed of lignocellulose and bio-based polymers. The bio-based polymers act as matrix materials, such as polylactic acid [6], polyvinyl alcohol [7], and thermoplastic starch [8]. The large number of hydroxyl groups in the anhydroglucose unit lead to poor interfacial adhesion between the lignocellulose and hydrophobic matrix. This results in the poor mechanical properties of the composites, because they have poor stress transfer properties. The water resistance of biocomposites is another important factor, which requires attention and should be addressed.

Epoxidized soybean oil (ESO) is a triglyceride and is widely used as a plasticizer in the plastics industry [9]. ESO has been investigated as a starting material for synthesizing polymer networks, by polymerization and crosslinking. Most ESO resin cannot be applied in commodities production, because of its poor mechanical properties compared with petroleum-based resins. This has been attributed to the low crosslinking density and flexible backbone structure of ESO [10]. Strategies to improve the properties of ESO resin have included incorporating clay [11] and natural fibers [12], and blending with epoxy resin [13].

ESO resin has recently been used as a modifier to improve the hydrophobicity of natural fibers, which has attracted broad interest. The ring opening of the epoxide in ESO reportedly leads to polymerization, initiated by hydroxyl groups and catalyzed by boron trifluoride diethyl etherate [14] or anhydrous stannic chloride [15], which has made a significant improvement to the water resistance and mechanical properties of lignocellulose fiber materials under wet conditions. In addition, the biodegradability of ESO resin cured with carboxylic acid and amine has been previously confirmed [16]. However, this polymerization process is cumbersome and is not eco-friendly, because it requires large solvent volumes, an inert atmosphere, or a catalyst. There are very few reports using ESO resin with polybasic carboxylic acid, polyphenol, and anhydride as curing agents, to modify natural fibers, because of their low curing efficiency. The renewable and environmental properties of curing agents have recently received increasing interest. Altuna et al. prepared a self-healing green ESO resin by cross-linking ESO in aqueous citric acid (CA) solution [17,18]. Gogoi et al. improved the mechanical properties of CA-cured ESO (ESOCA) resin by incorporating carboxylic acid-functionalized multiwalled carbon nanotubes [19]. Shibata et al. prepared ESO resin by curing with tannic acid, and improved the mechanical properties by incorporating microfibrillated cellulose into the ESO resin [20]. However, the curing conditions still require improving, particularly by lowering the curing time and temperature, which will promote the practical application of the resulting materials.

**Scheme 1:**
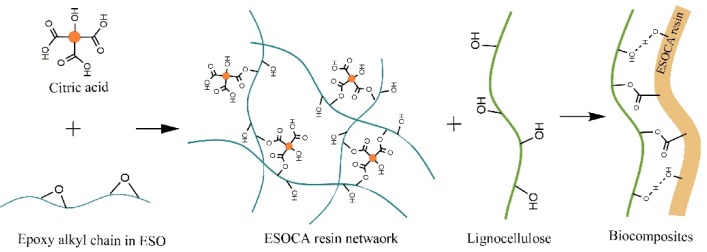
Schematic of lignocellulose modified by epoxidized soybean oil (ESO) resin cured with citric acid.

We were inspired to make better use of ESO resin with poor mechanical properties, by using it to modify lignocellulose materials. The approach is based on the amphipathicity of ESO resin, which results from its non-polar alkyl chain, as well as polar hydroxyl groups and carboxylic groups (Scheme 1). Specifically, we prepared lignocellulose biocomposites by modifying lignocellulose with ESO resin cured by CA, to obtain biocomposites that are renewable, biodegradable, and exhibit high hydrophobicity and tensile strength. We aimed to efficiently utilize the lignocellulose and plant oil resources, which will promote the application of the resulting biocomposites in packaging. The thermal stability, curing efficiency, micromorphology, tensile strength, water contact angle, and water absorption properties of the biocomposites were characterized.

## 2. Materials and Methods

### 2.1. Materials

Sisal fiber (SF) was supplied by Shenzhen Xian’gu Tech Co. Ltd. (Shenzhen, China). ESO (average molecular weight = 974 Da; average functionality = 6 epoxides per triglyceride) was purchased from Shanghai Aladdin Biochemical Technology Co. Ltd. (Shanghai, China) CA, and tetrahydrofuran was purchased from Sinopharm Chemical Reagent Co. Ltd. (Shanghai, China). All reagents were of analytical grade and were used without purification.

### 2.2. Preparation of Steam Exploded Sisal Fiber (SESF) Preforms

SF was first pretreated with continuous screw extrusion steam explosion (SESE), according to a reported method [21]. Briefly, SF was cut into short fibers with lengths of approximately 10 mm. The screw continuously conveyed the SF chips with about 50% moisture content forward, where they were squashed and compacted by the screw, and gradually heated due to friction between the chips, screw, and barrel. The pressure and temperature of the compacted SF reached approximately 1.5 MPa and 150 °C, respectively, when they conveyed to the die. The compacted SF was continuously discharged from the die, with a slit width of 1 mm. Pressurized water in the fiber bundles was instantaneously vaporized, which resulted in destruction of the tissue structure of the fiber bundles. This pretreatment process was repeated three times to obtain SESF.

Two grams of SESF were then immersed in 100 mL of water, which was subjected to high speed stirring for 2 min to disperse the SESF. The wet filter cake was obtained by filtering with a Buchner funnel, and was then dried at 105 °C in an oven to obtain the SESF preforms.

### 2.3. Preparation of ESOCA Prepolymer

Three parts of CA (by weight) were completely dissolved in one part of distilled water at 90 °C. ESO was then added, and dispersed uniformly with magnetic stirring. Carboxylic acid to epoxy group molar ratios (R) of 0.8, 1.0, 1.2 and 1.5 were obtained, by varying the amount of ESO that was added. The mixture was heated to 90 °C and stirred for 15 min to form a homogeneous solution. The mixture was then cooled to room temperature. A certain volume of tetrahydrofuran was poured into the bottle to obtain an ESOCA prepolymer solution with a concentration of 0.2 g/mL.

### 2.4. Preparation of SESF/ESOCA Resin (SEC) Biocomposites

**Scheme 2:**
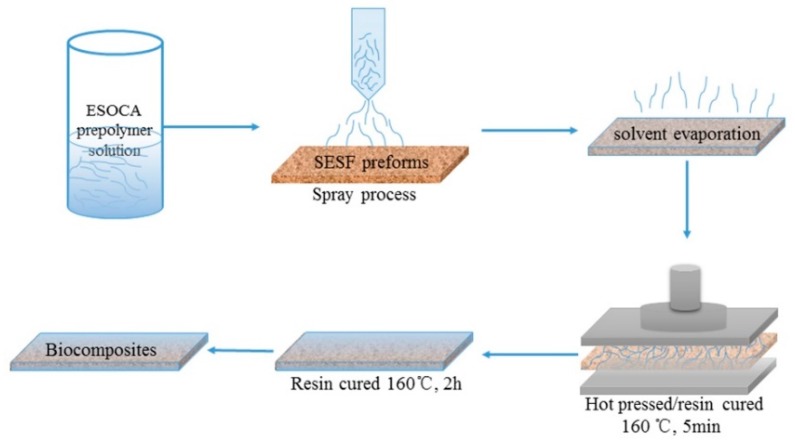
The preparation procedure of SESF/ESOCA (SEC) biocomposites.

Various volumes of ESOCA prepolymer solution were sprayed into the SESF preforms, and the solvent was then volatilized in a fuming cupboard. The SESF preforms containing ESOCA prepolymer were cured for 5 min at 160 °C at a pressure of 2 MPa, in a mold with a height of 1 mm. The resulting biocomposites were further cured for 2 h at 160 °C at atmospheric pressure in an oven. The weight percentage of ESOCA resin in the composites were controlled at 20%, 30%, 40% or 50%. The resulting composites are denoted SECR/y, where R is the carboxylic acid to epoxy group molar ratio (0.8, 1, 1.2 or 1.5), and y is ESOCA resin content (0, 20%, 30%, 40%, 50% or 100%). SECR/0 and SECR/100 corresponded to SESF preforms and pure ESOCAR resin, respectively. The preparation process of biocomposites is shown in Scheme 2. The abbreviated names of samples obtained after various stages of treatment are shown in Table 1.

### 2.5. Characterization

#### 2.5.1. Fourier-Transform Infrared (FT-IR) Spectroscopy

FT-IR spectra were recorded using a Nexus670 FT-IR spectrometer (Thermo Nicolet, Waltham, MA, USA), with a resolution of 4 cm^−1^ and scanning range of 4000~500 cm^−1^. The data were processed and analyzed with OMNIC analysis software.

#### 2.5.2. Thermogravimetric Analysis (TGA)

The TGA of these materials was performed using a 209F3 thermal analyzer (NETZSCH, Selb, Germany), under a N_2_ atmosphere with a N_2_ flow rate of 50 mL/min. The heating rate was 10 °C/min, and the scanning range was 30~700 °C.

#### 2.5.3. Differential Scanning Calorimetry (DSC)

The curing efficiency of ESOCA resin in the biocomposites was measured using a DSC 240 F1 apparatus (NETZSCH, Selb, Germany). Approximately 5 mg of sample was placed in an aluminum DSC pan. Thermal scanning was then carried out from −50 °C to 150 °C, at a heating rate of 10 °C/min under a N_2_ atmosphere.

#### 2.5.4. Mechanical Properties

Tensile testing was performed using an Instron 5566 universal testing machine (Instron, Norwood, MA, USA) with a 50 N load cell, at a controlled temperature of 23 °C and relative humidity of 50%. Strips with widths of 5 mm and heights of 1 mm were clamped with a free span of 20 mm, and were subjected to strain at a constant rate of 5 mm/min. Five mechanical tests were performed for each material, and the values were averaged. Stress–strain curves were recorded, from which the tensile strength was obtained.

#### 2.5.5. Microtopography

The surface and cross section of the composites were observed by field emission scanning electron microscopy, using a 1530 VP microscope (Zeiss, Jena, Germany). The operating voltage was 5 kV. Prior to analysis, samples were adhered to specimen holders using double-sided conductive adhesive tape, and then coated with gold to produce a 10-nm-thick film on the sample surface.

#### 2.5.6. Water Absorption Measurements

Strips were immersed in water for various times, then removed and gently wiped to remove residual water from the surface. The weight of each material was recorded using five parallel tests. The water absorption was calculated according to the equation: water absorption = (Wi/Wc − 1) × 100%, where Wc and Wi are the weight of the strips before and after immersion, respectively. The water absorption was averaged based on five measurement results.

#### 2.5.7. Water Contact Angle Measurements

The contact angles of water droplets on the sample surfaces were measured at room temperature, using an OCA40 Micro geniometer (Dataphysics, Filderstadt, Germany). Five microliters of deionized water were placed on the sample surface. Five contact angle measurements were recorded at different points for each sample, from which the average contact angle was determined.

## 3. Results and Discussions

### 3.1. Effect of SESE Pretreatment on Morphology of SF

Each individual SF is a multicellular bundle of polygonal hollow sub-fibers [22]. These SF bundles are rigid and have widths of 50~100 μm. These properties make it difficult to curve and mutually entangle, which causes a problem when preparing non-woven natural fiber preforms and composites. Some researchers have therefore prepared natural fiber preforms using bacterial cellulose and cellulose nanofiber as binders, to improve their mechanical properties [23].

**Figure 1:**
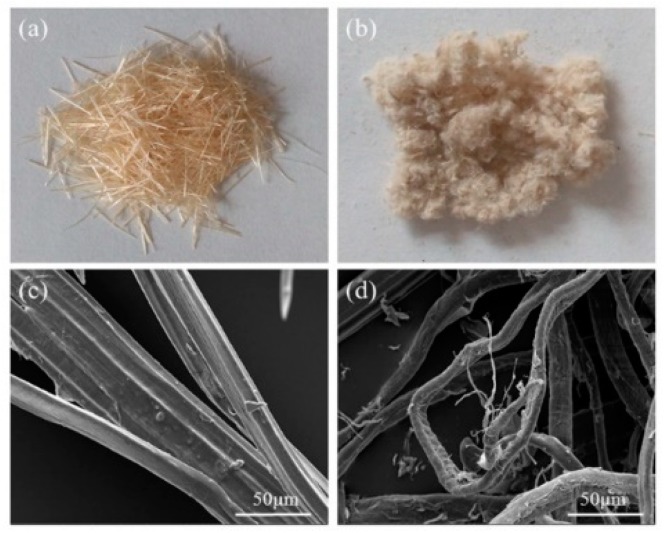
Digital photographs and SEM images of fibers, before screw extrusion steam explosion (SESE) pretreated (**a**,**c**) and after SESE pretreatment (**b**,**d**).

In the current study, the SF bundle structure was destroyed by the screw extrusion steam explosion pretreatment. The resulting SESF had a much smaller diameter of 10~20 μm, and an aspect ratio of 200~500. Figure 1 shows that the SESF had a flocculent structure. This resulted from the mutually entangled SESF, that had a high aspect ratio, and differed to the bundled structure of SF. The obtained SESF, that had a low diameter, high aspect ratio and high specific area, is favorable for the preparation of SESF non-woven preforms and composites. Then, the exposure of polysaccharide has previously confirmed [24], which resulted in many more hydroxyl groups on the surface of SESF. This is beneficial to the performance of materials obtained in this work. Therefore, the SESE pretreatment was chosen to prepare natural fiber preforms and composites.

### 3.2. FT-IR Spectroscopy

**Figure 2:**
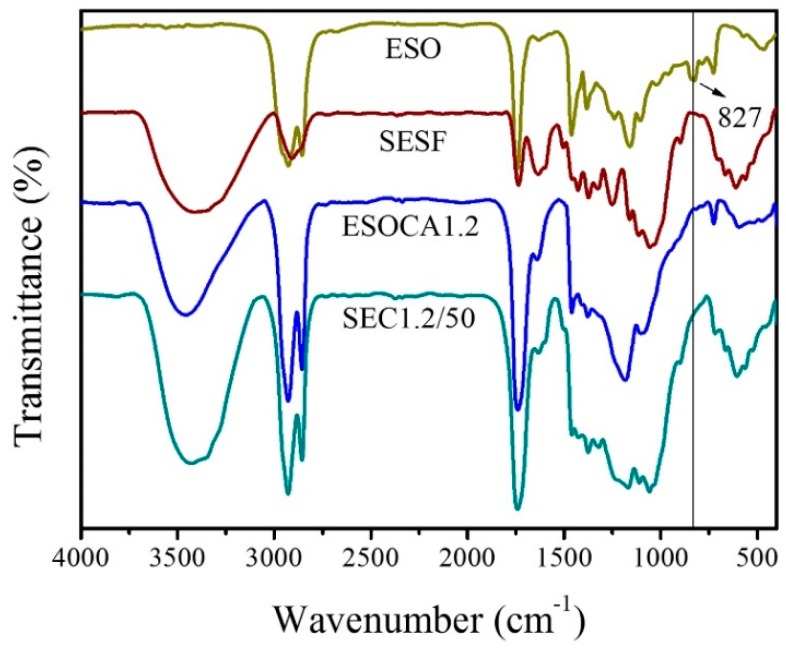
Fourier-transfer infrared spectroscopy (FT-IR) spectra of epoxidized soybean oil (ESO), steam exploded sisal fiber (SESF), CA-cured ESO Resin with R =1.2 (ESOCA1.2), and SESF/ESOCA1.2/50 biocomposites (SEC1.2/50).

FT-IR spectra of SESF, ESO, ESOCA resin, and SEC biocomposites are shown in Figure 2. The peak at 3447 cm^−1^ in the spectrum of ESOCA resin corresponded to hydroxyl groups, which resulted from the reaction between epoxy and carboxylic acid groups. The peak at 827 cm^‒1^ in the spectrum of ESO corresponded to epoxy groups, and was absent in the spectra of ESOCA resin and SEC biocomposites. This indicated that the epoxy groups had been consumed in the ring opening polymerization reaction, with citric acid in aqueous solution. The intensity of the peak at 1736 cm^−1^ in the spectra of ESOCA resin and SEC biocomposites was higher than that of SESF, because of the formation and addition of ester bonds. The peaks at 2928 cm^−1^ and 2853 cm^−1^ corresponded to methyl and methylene units, respectively. The increase in their intensities in the spectrum of SEC biocomposites was attributed to the addition of alkyl chains. The alkyl chains were projected to the surface, which promoted the hydrophobicity of SEC biocomposites.

### 3.3. TGA

**Figure 3:**
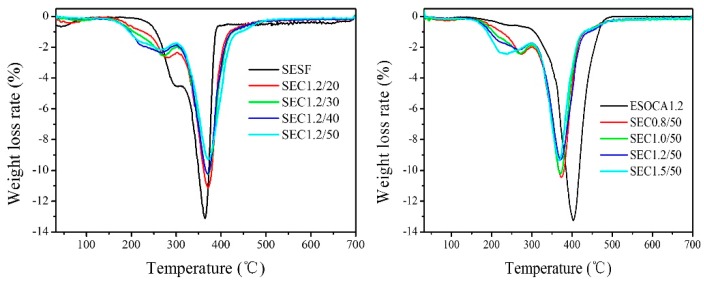
Differential thermogravimetric (DTG) curves of SEC biocomposites with different R values and ESOCA content.

The thermal stabilities of SEC biocomposites with different R values and ESOCA resin contents were evaluated by TGA, as shown in Figure 3. The first differential thermogravimetric (DTG) curve of SESF showed a shoulder peak at approximately 300 °C, and a sharp peak at 364.1 °C. These represented the maximum degradation temperatures of hemicellulose and cellulose, respectively. The maximum degradation temperature of pure ESOCA resin was approximately 400 °C. The DTG curves of SEC biocomposites showed that the peaks of the maximum degradation temperatures of ESOCA resin and SESF approached each other, to appear as a single peak at approximately 373.5 °C. This indicated that the addition of ESOCA resin, with its high thermostability, had a ‘protect and delay’ effect on the thermal degradation of SESF. It also suggested that there was good compatibility between SESF and ESOCA resin, because of the presence of polar hydroxyl and carboxylic groups. The weak shoulder peak at 200~250 °C, in the curve of ESOCA resin, may be due to the decomposition of low-molecular-weight components, such as CA curing agent [25]. There was a small shift of the broad peak, from about 300 °C to about 270~280 °C, which may be due to hemicellulose degrading more readily under acidic conditions [26]. Increasing the R value and ESOCA resin content would increase the rate of hemicellulose degradation. There was a small shoulder peak after 400 °C, which was attributed to the thermal degradation of a few ESO without reaction.

### 3.4. DSC

**Figure 4:**
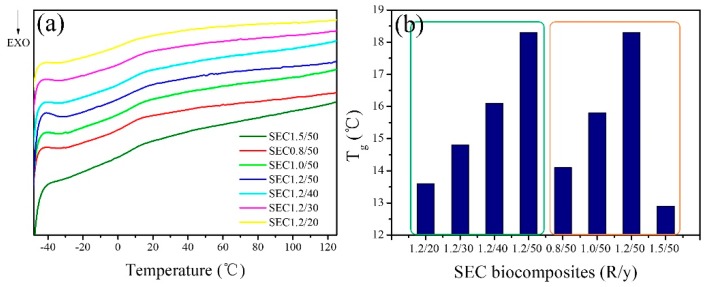
DSC curves (**a**) and T_g_ values (**b**) of SEC biocomposites with different R values and ESOCA resin contents.

The curing efficiency of ESOCA prepolymer in the SESF preforms was investigated by DSC (Figure 4a). The DSC results reflected the crosslinking degree of ESOCA resin, which reportedly affects the performance of SEC biocomposites [27]. Figure 4b shows that the glass transition temperature (T_g_) of ESOCA resin in SEC biocomposites gradually increased from 13.6 °C (SEC1.2/20) to 18.3 °C (SEC1.2/50) with increasing ESOCA resin content. The main reason for this was because ESOCA prepolymer was uniformly dispersed in the SESF preforms. At low ESOCA resin content, there was less contact between ESOCA resin and SESF preforms, and thus less potential for curing. A high ESOCA resin content yielded better curing efficiency and a higher crosslinking degree, resulting in a higher T_g_. The R value also significantly affected the curing efficiency. T_g_ of ESOCA increased from 14.1 °C to 18.3 °C as the R value increased from 0.8 to 1.2, and then decreased to 12.9 °C at R = 1.5. A slight increase in the R value reported improved the curing efficiency and crosslinking degree [17]. However, a high R value resulted in a decrease in curing efficiency. This was largely attributed to most epoxy groups reacting with a single CA molecule, so there were few epoxy groups in ESOCA prepolymer to participate in further curing reactions.

### 3.5. Mechanical Properties of the Biocomposites

**Figure 5:**
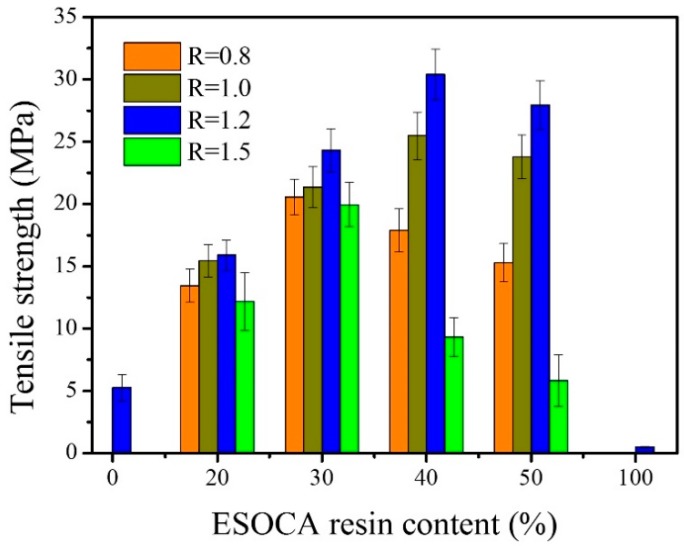
Tensile strength of SEC biocomposites with different R values and ESOCA resin content.

Tensile strength of SEC biocomposites are shown in Figure 5. The tensile strength of the SESF preforms and ESOCA resin with R = 1.2 were 5.26 MPa and 0.49 MPa, respectively. The tensile strength of SEC biocomposites was significantly improved, compared with that of the SESF preforms and ESOCA resin. Tensile strength of SEC biocomposites was affected by R value, for a constant ESOCA resin content. The maximum and minimum tensile strengths of SEC biocomposites were observed for R = 1.2 and R = 1.5, respectively. The trend in tensile strength corresponded with the curing efficiency of ESOCA resin, which was observed from the DSC results.

On the other hand, tensile strength first increased and then decreased with increasing ESOCA resin content, for a constant R value. The optimum ESOCA resin content in the current study was found to vary depending on the R value. For example, the optimum ESOCA resin content was 30% for SEC0.8/y and SEC1.5/y, but was 40% for SEC1.0/y and SEC1.2/y. The reason for this could be that ESOCA resin with low curing efficiency had worse mechanical properties, and thus negatively affected the tensile strength with increasing ESOCA resin content. For all SEC biocomposites, SEC1.2/40 exhibited the highest tensile strength of 30.4 MPa, which was much higher than those of the SESF preforms and ESOCA resin.

### 3.6. Morphologies of SEC Biocomposites

**Figure 6:**
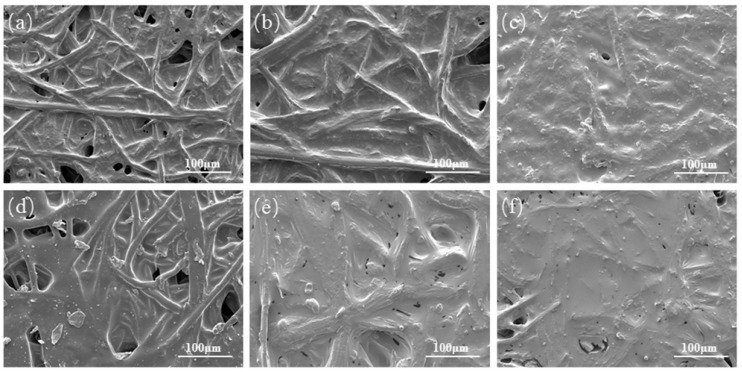
SEM images showing the surface morphologies of (**a**) SEC1.2/30, (**b**) SEC1.2/40, (**c**) SEC1.2/50, (**d**) SEC0.8/40, (**e**) SEC1/40, and (**f**) SEC1.5/40.

**Figure 7:**
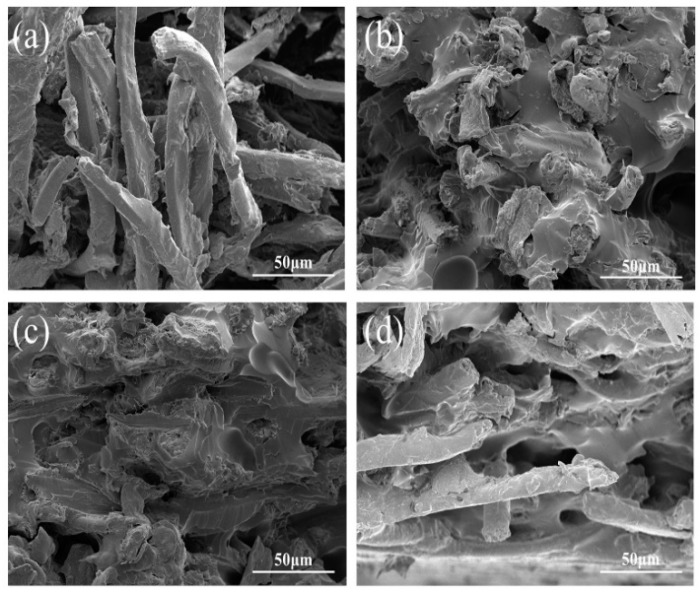
SEM images of cross section morphology of (**a**) SEC1.2/20, (**b**) SEC1.2/40, (**c**) SEC1.2/50, and (**d**) SEC1.5/50.

The morphologies of SEC biocomposites, with different R values and ESOCA resin contents, were observed by SEM. SEM images of the surface and cross section of SEC biocomposites are shown in Figure 6 and Figure 7, respectively. Figure 6a–c shows that increasing ESOCA resin content resulted in fewer holes in the surface of SEC biocomposites, and also resulted in ESOCA resin phase being more continuous. This resulted in SESF being better coated and bound by ESOCA resin. ESOCA resin with different R values resulted in different surface morphologies of SEC biocomposites. As shown in Figure 6b,d–f, the profile of SESF in ESOCA resin was more clearly observed on the surfaces of SEC biocomposites with R = 1.0 or 1.2. This may have been attributed to the more efficient curing process of ESOCA resin, resulting in more shrinkage around the SESF.

SEM images of cross sections of SEC biocomposites are shown in Figure 7. These revealed the fracture mechanisms of biocomposites with different R values and ESOCA resin contents. According to Figure 7a, ESOCA resin continuous phase was not obvious. The SESF cannot be wetted and bonded by ESOCA resin adequately. Therefore, the slippage and separation of SESF occurred easily in the process of mechanical stretch, and it was difficult to get a favorable stress transfer in the interface between fibers and resin, as resin can be destroyed easily. There were significant differences in cross section morphology of the SEC1.2/40 biocomposites compared with that of the SEC1.2/20 biocomposites. As shown in Figure 7b, SESF fractured in the cross section of SEC biocomposites, and lacerated ESOCA resin was well coated on the SESF surface. This indicated that ESOCA resin could provide favorable stress transfer in SEC biocomposites because of excellent interface bonding between SESF and ESOCA resin [28]. For the high ESOCA resin content in Figure 7c, SESF mainly fractured along the cross section of ESOCA resin. It was difficult to observe the destruction of the interface between SESF and ESOCA resin. This was because a SESF fracture was followed by ESOCA resin being quickly destroyed due to its poor mechanical performance. This material therefore had inferior stress transfer properties, and thus would more quickly fail during application.

The cross section of SEC1.5/50 biocomposites is shown in Figure 7d. Holes were observed in the cross section, as a result of SESF being mechanically pulled away. ESOCA resin with low curing efficiency could not provide satisfactory stress transfer, and resulted in the fracture of SESF. These SEM images revealed the fracture mechanisms of SEC biocomposites with different ESOCA resin contents and R values, which were also corresponding with the change of tensile strength of SEC biocomposites.

### 3.7. Water Absorption

**Figure 8:**
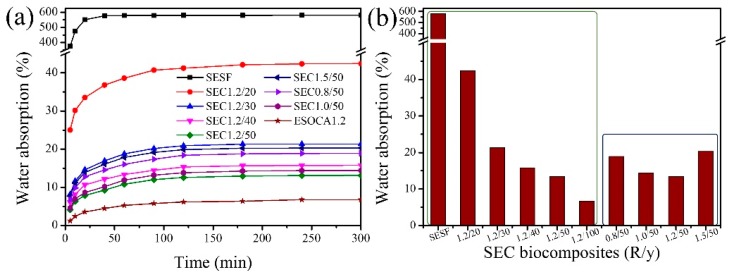
Water absorption of SEC biocomposites with different R values and ESOCA resin content, (**a**) water absorption depending on time, (**b**) maximum water absorption.

Water resistance of SEC biocomposites is important for their practical application, and was investigated by carrying out water absorption measurements. The water absorption of different samples, depending on time and the maximum water absorption, are presented in Figure 8a,b, respectively. The results indicated that the presence of ESOCA resin significantly decreased water absorption of SEC biocomposites. Water absorption of SESF was 580.6%, which decreased with increasing ESOCA resin content, reaching a minimum of 13.1% for the SEC1.2/50 biocomposites. The main reason for this was that higher ESOCA resin content coated SESF more completely. Higher ESOCA resin content also decreased the interspace in SEC biocomposites, which prevented water penetrating into SESF in SEC biocomposites. SEC biocomposites with different R values also had different water absorption. SEC biocomposites containing high curing efficiency ESOCA resin showed high water resistance, which was due to the good interfacial bonding between SESF and ESOCA resin. Thus, the SEC1.0/50 and SEC1.2/50 biocomposites had relatively low water absorption of 15.7% and 13.1%, respectively.

### 3.8. Water Contact Angles of SEC Biocomposites

**Figure 9:**
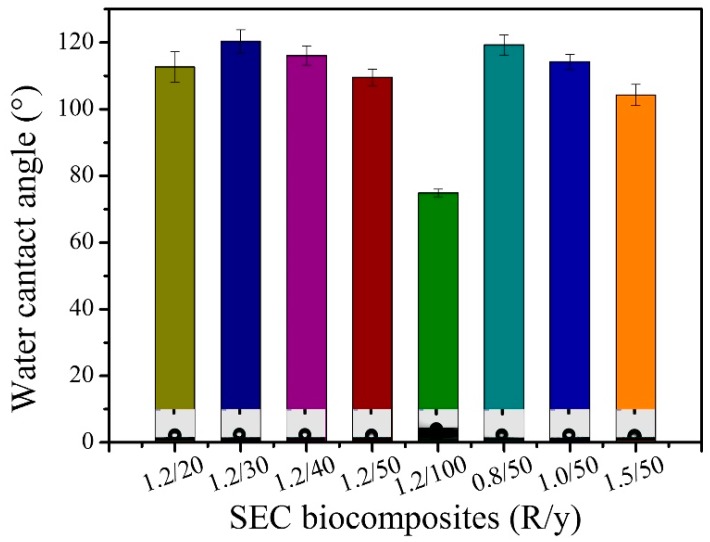
Water contact angles of SEC biocomposites with different R values and ESOCA resin content.

**Scheme 3:**
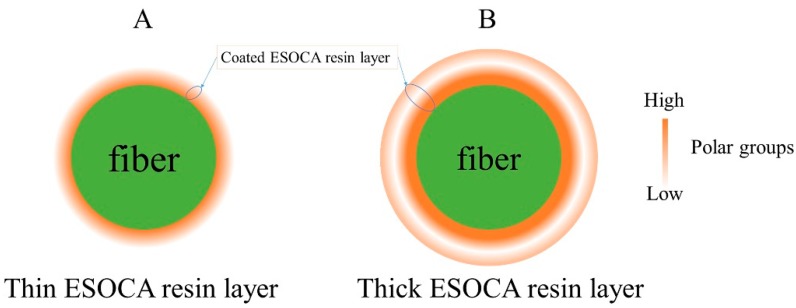
Distributions of polar groups in ESOCA resins with different thicknesses.

The water contact angle of the SESF preforms was not measured because of complications arising from its high hydrophilicity and porosity. The water contact angle of ESOCA1.2 was 74.9°, as shown in Figure 9. This indicated that the surface of ESOCA resin was hydrophilic. However, SEC biocomposites still exhibited low water absorption, as the long hydrophobic alkyl chains of ESOCA resin prevented significant amounts of water from entering [29]. There was a significant improvement in water contact angle of all SEC biocomposites, compared with that of the SESF. This was attributed to polar groups (hydroxyl and carboxyl groups) combining with hydroxyl groups on the SESF surface, and also to more hydrophobic groups being exposed to the surface of ESOCA resin. These results suggested that the water contact angle was effected by ESOCA resin content. Furthermore, the addition of ESOCA resin also aided surface roughness, which would increase the water contact angle under the condition of low polar groups content [30]. An appropriate ESOCA resin content (30%) completely coated the SESF, forming a relatively thin resin layer. This preferentially orientated the polar groups towards the SESF surface, as shown in Scheme 3A. When ESOCA resin content was high, the ESOCA resin layer was much thicker. In this situation, the content of surface polar groups approached that of the pure ESOCA resin, as shown in Scheme 3B. Additionally, the surface of biocomposites were gradually smoothed. This resulted in a slight decrease in water contact angle. Therefore, the maximum water contact angle of 120.4° was observed for the SEC1.2/30 biocomposites. The effect of the R value on the water content angle was also investigated at an ESOCA resin content of 50%. The SEC0.8/50 biocomposites had a maximum water contact angle of 119.2°, which gradually decreased with increasing R. The main reason for this was that the number of surface polar groups in the SEC biocomposites increased with increasing R, which resulted in the decrease of water contact angle.

## 4. Conclusions

Continuous screw extrusion steam explosion was used to pretreat SF, in which the bundled structure of SF was destroyed, yielding SESF with a high aspect ratio. This finding is significant for the general application of SF in various fields. Amphiphilic ESOCA resin exhibited excellent interfacial bonding with the SESF. The mechanical properties of the resulting SEC biocomposites were significantly improved compared with those of SESF, due to their favorable stress transfer properties. Tensile strength of the SEC1.2/40 biocomposites was 30.4 MPa. SEC biocomposites exhibited favorable water resisting properties and water contact angles, which is important to their application in humid environments. An appropriate R value and high ESOCA resin content resulted in a high curing degree. ESOCA resin with high curing possess high mechanical properties and densification, which can result in favorable bonding strength between fibers, and inhibit the permeation of water into SEC composites. It is beneficial to improve mechanical properties and water resistance. In addition, ESOCA resin content has an important effect on the coating of fibers. High ESOCA resin content can result in more complete bonding between fibers, and a decrease of holes in SEC biocomposites, which is also favorable to mechanical properties and water resistance. However, exorbitant ESOCA resin content is detrimental to mechanical properties and water contact angle. Lignocellulose and ESO resin with poor mechanical properties can therefore be used to efficiently prepare biocomposites with excellent mechanical performance. This will promote the development of biocomposites, and ease resource demands and environmental problems.

## Figures and Tables

**Table 1:** The abbreviated names of different samples.

Abbreviations	Name
ESO	Epoxidized Soybean Oil
CA	Citric Acid
ESOCA	CA-Cured ESO Resin
SESE	Screw Extrusion Steam Explosion
SF	Sisal Fiber
SESF	Steam Exploded Sisal Fiber
SEC	SESF/ESOCA Biocomposites
